# BDP1 Alterations Correlate with Clinical Outcomes in Breast Cancer

**DOI:** 10.3390/cancers14071658

**Published:** 2022-03-25

**Authors:** Stephanie Cabarcas-Petroski, Laura Schramm

**Affiliations:** 1 Department of Biology, Beaver Campus, Pennsylvania State University, Monaca, PA 15061, USA; smc1088@psu.edu; 2 Department of Biology, St. John’s University, Queens, NY 11439, USA

**Keywords:** BDP1, TFIIIB, BRF2, BRF1: RNA polymerase III, breast cancer

## Abstract

**Simple Summary:**

Breast cancer accounts for 30% of all new cancer diagnoses in the United States. The most common type of breast cancer is invasive breast cancer. A hallmark trait of breast cancer is uncontrolled cell growth due to genetic alterations. TFIIIB-mediated RNA polymerase III transcription is specifically deregulated in human cancers. The TFIIIB BDP1 subunit is not well characterized in human cancer. The objective of this study was to analyze publicly available clinical cancer datasets to determine if BDP1 alterations correlate with clinical outcomes in available breast cancer datasets. BDP1 copy number and expression negatively correlate with breast cancer outcomes, including stage, grade, and mortality.

**Abstract:**

TFIIIB is deregulated in a variety of cancers. However, few studies investigate the TFIIIB subunit BDP1 in cancer. BDP1 has not been studied in breast cancer patients. Herein, we analyzed clinical breast cancer datasets to determine if BDP1 alterations correlate with clinical outcomes. BDP1 copy number (*n =* 1602; *p =* 8.03 × 10^−9^) and mRNA expression (*n =* 130; *p =* 0.002) are specifically decreased in patients with invasive ductal carcinoma (IDC). In IDC, BDP1 copy number negatively correlates with high grade (*n =* 1992; *p =* 2.62 × 10^−19^) and advanced stage (*n =* 1992; *p =* 0.005). BDP1 mRNA expression also negatively correlated with high grade (*n =* 55; *p =* 6.81 × 10^−4^) and advanced stage (*n =* 593; *p =* 4.66 × 10^−4^) IDC. Decreased BDP1 expression correlated with poor clinical outcomes (*n =* 295 samples): a metastatic event at three years (*p =* 7.79 × 10^−7^) and cancer reoccurrence at three years (*p =* 4.81 × 10^−7^) in IDC. Decreased BDP1 mRNA correlates with patient death at three (*p =* 9.90 × 10^−6^) and five (*p =* 1.02 × 10^−6^) years. Both BDP1 copy number (*n =* 3785; *p =* 1.0 × 10^−14^) and mRNA expression (*n =* 2434; *p =* 5.23 × 10^−6^) are altered in triple-negative invasive breast cancer (TNBC). Together, these data suggest a role for BDP1 as potential biomarker in breast cancer and additional studies are warranted.

## 1. Introduction

In the United States (U.S.), breast cancer accounts for 30% of new cancer diagnoses in women [1]. Breast cancer incidence rates continue to increase approximately 0.5% each year, with breast cancer being the leading cause of death in women aged 20 to 59 [1]. Breast cancers are classified by site and if the breast cancer is invasive or non-invasive [2]. Approximately 287,850 new cases of invasive breast cancer (IBC) and 51,400 cases of non-invasive breast cancer are expected to be diagnosed in 2022 [3]. The most common types of breast cancer include ductal carcinoma in situ (DCIS), invasive ductal carcinoma (IDC), and invasive lobular carcinoma (ILC) [2]. Together, IDC and ILC account for 90% of all IBC; DCIS is the most common non-invasive breast cancer diagnosed [4].

Many cancers, including breast cancer, are driven by genetic and epigenetic alterations leading to deregulated cell proliferation. In eukaryotes, cell proliferation is regulated, in part, by three RNA polymerases (pol) [5]. RNA pol I transcribes genes encoding ribosomal RNA required by ribosomes; RNA pol II transcribes mRNA encoding proteins and some small untranslated RNA molecules involved in RNA processing [5]. RNA pol III transcribes untranslated RNA molecules involved in processing and translation [5]. Together, RNA pol I and pol III regulate the biosynthetic capacity of a cell.

It is well-established that RNA pol III transcription is deregulated in various human cancers [6,7]. The initiation of RNA pol III transcription requires TFIIIB [5,8]. Two forms of TFIIIB have been identified in higher eukaryotes [8,9]. Accurate transcription initiation from gene-internal RNA pol III promoters requires a TFIIIB complex containing TBP, BRF1, and BDP1 [8,9]. Transcription from gene-external RNA pol III promoters requires a TFIIIB complex containing TBP, BRF2, and BDP1 [8,9]. TFIIIB activity is inhibited by tumor suppressors, including p53 [10,11], PTEN [12,13,14], BRCA1 [15], the retinoblastoma protein (Rb) [11], and the Rb family members p107 and p130 [16]. The oncogenes MAP kinase ERK and MYC [11,17] stimulate TFIIIB activity in vitro. The TFIIIB subunits TBP [18,19,20], BRF1 [21,22,23,24], and BRF2 [6,22,25,26,27,28,29,30,31,32] have begun to be studied in specific human cancers. For example, BRF2 has been classified as a novel proto-oncogene in human cancers [6,27,31,32]. However, studies that determine whether BDP1 is altered in human cancers and clinically relevant are limited. A study implicating BDP1 in prostate cancer was performed in a PTEN-null prostate cancer cell line [14]. Somatic frameshift mutations in BDP1 have been identified in colorectal cancer, *n =* 98, but clinical outcome data were not reported in the commentary [33]. Recently, BDP1 expression has been correlated with clinical outcomes in non-Hodgkin lymphoma [34]. Together, these recent studies suggest a detailed analysis of BDP1 alterations in human cancers is warranted.

The objective of this study was to analyze publicly available clinical cancer datasets to determine if BDP1 alterations correlate with clinical outcomes in available breast cancer datasets. Herein, we report that BDP1 copy number (*n =* 1602; *p =* 8.03 × 10^−9^) and mRNA expression (*n =* 130; *p =* 0.002) are specifically decreased in patients with invasive ductal carcinoma (IDC). In IDC, BDP1 copy number negatively correlated with high grade (*n =* 1992; *p =* 2.62 × 10^−19^) and advanced stage (*n =* 1992; *p =* 0.005). BDP1 mRNA expression also negatively correlated in high grade (*n =* 55; *p =* 6.81 × 10^−4^) and advanced stage (*n =* 593; *p =* 4.66 × 10^−4^) IDC. Interestingly, decreased BDP1 expression correlated with clinical outcomes (*n =* 295 samples): a metastatic event at three years (*p =* 7.79 × 10^−7^) and cancer reoccurrence at three years (*p =* 4.81 × 10^−7^) in patients with invasive breast cancer (IBC). Decreased BDP1 mRNA correlates with patient death at three (*p =* 9.90 × 10^−6^) and five (*p =* 1.02 × 10^−6^) years. BDP1 copy number decreased in triple-negative invasive breast cancer (TNBC) (*n =* 3786; *p =* 1.04 × 10^−21^). Both BDP1 copy number (*n =* 3785; *p =* 1.0 × 10^−14^) and mRNA expression (*n =* 2434; *p =* 5.23 × 10^−6^) are altered in TNBC. Additionally, BDP1 mRNA expression is increased by the breast cancer chemotherapeutics doxorubicin (13.146-fold increase; *p =* 4.43 × 10^−4^), etoposide (9.703-fold increase; *p =* 8.15 × 10^−4^), fluorouracil (9.468-fold increase; *p =* 0.005), and bortezomib (1.831-fold increase; *p =* 0.002) in well-studied breast cancer cell lines [35,36]. Taken together, these data suggest a role for BDP1 alterations in invasive breast cancer. Additional studies are warranted to determine if BDP1 may be a novel target for breast cancer therapy.

## 2. Materials and Methods

BDP1 copy number and mRNA expression in breast cancer were analyzed using microarray datasets available in the Oncomine^TM^ Research Edition Platform [37,38]. The Oncomine^TM^ Research Premium Edition Platform is a cancer microarray database and web-based data-mining platform [37,38] containing 729 cancer datasets (91,866 samples) and was queried, January 2020–January 2022, to determine if BDP1 alterations’ in breast cancers correlate with clinical outcomes [37,38]. The Oncomine^TM^ Research Premium Edition Platform datasets are log-transformed, median centered per array, and standard deviation normalized to one per array; statistical tests conducted both as two-sided for differential expression analysis and one-sided for specific over- and under-expression analysis [37,38]. For whole study analysis, *p*-values were corrected for multiple comparisons by the method of false discovery rates (FDR) [37,38].

For BDP1 expression analyses in specific datasets, cut-off values, sample numbers, and *p*-values are indicated in the figure legends. The Oncomine™ Platform (Thermo Fisher, Ann Arbor, MI, USA) was used for analysis and visualization. Public datasets queried are noted below, with hyperlinks to the available datasets and study descriptions (Table 1) and are cited in figure legends.

## 3. Results

We queried all breast cancer datasets housed in the Oncomine^TM^ Research Platform (132 datasets, 14,277 samples) [37,38] to determine if BDP1 copy number or mRNA expression is altered in breast cancer and if BDP1 alterations correlated with clinical outcomes in breast cancer.

### 3.1. BDP1 Copy Number and mRNA Expression Is Decreased in IDC

**Figure 1:**
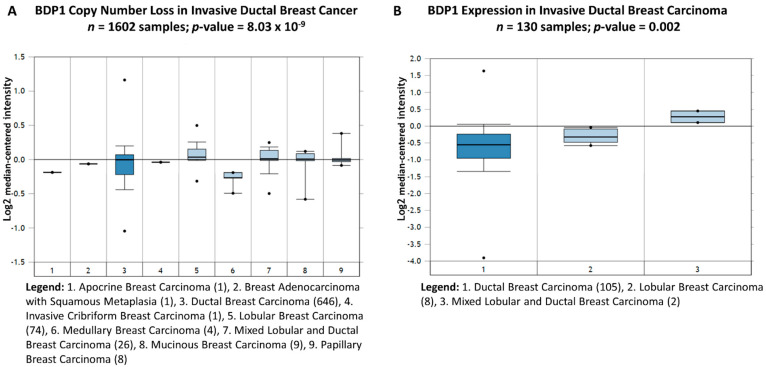
BDP1 DNA copy number and mRNA expression decrease in invasive ductal carcinoma (IDC). (**A**) We queried the TCGA breast dataset [45,46,47], *n*  = 1602 samples, and BDP1 copy number is significantly decreased in IDC (−1.073-fold change), *p*-value = 8.03 × 10^−9^. The gene rank for BDP1 DNA copy number loss in IDC is in the top 1% (139) of 18,823 measured genes in the TCGA breast dataset [45,46,47]. (**B**) BDP1 mRNA expression is significantly decreased in IDC (−1.341-fold change) when querying the Esserman breast cancer dataset [41], *n* = 130 samples, *p*-value = 0.002. The gene rank for BDP1 mRNA expression in IDC is in the top 1% (72) of 19,189 measured genes in the Esserman dataset [41]. Bright blue denotes statistically significant BDP1 changes. The Oncomine™ Platform (Thermo Fisher, Ann Arbor, MI, USA) was used for analysis and visualization.

A query of the 132 breast cancer datasets in the Oncomine^TM^ Research Edition Platform [37,38] for BDP1 alterations identified changes in BDP1 copy number and mRNA expression in invasive ductal carcinoma (IDC) in cancer versus cancer histology analysis, Figure 1. Ductal carcinoma is classified as non-invasive ductal carcinoma in situ (DCIS) or invasive ductal carcinoma (IDC). Annually, IDC is the most frequently diagnosed form of breast cancer. BDP1 copy number is significantly decreased in IDC (−1.073-fold change, *p* = 8.03 × 10^−9^) in the TCGA breast cancer dataset (*n =* 1602) [45,46,47], Figure 1A. The gene rank for BDP1 DNA copy number loss in IDC is in the top 1% (139) of 18,823 measured genes in the TCGA breast dataset [45,46,47]. Figure 1B demonstrates that BDP1 mRNA expression is significantly decreased in IDC (-1.341-fold change, *p* = 0.002) in the Esserman breast cancer dataset (*n* = 130) [41]. The gene rank for BDP1 in IDC is in the top 1% (72) of 19,189 measured genes in the Esserman breast cancer dataset [41].

### 3.2. BDP1 Alterations Negatively Correlate in High Grade and Advanced Stage IDC

**Figure 2:**
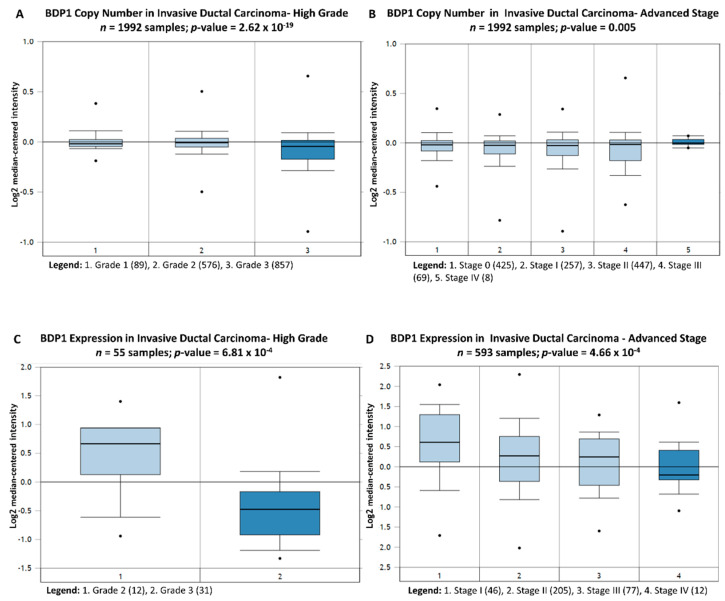
BDP1 copy number and mRNA expression negatively correlate with advanced grade and stage in invasive ductal carcinoma (IDC). (**A**) BDP1 copy number is significantly decreased in the Curtis breast dataset [40], *n* = 1992 samples and *p*-value = 2.62 × 10^−19^. The gene rank for BDP1 under-expression is in the top 1% (66) of 18,823 measured genes in IDC, high grade. Using the same Curtis Breast dataset [40], (**B**) BDP1 copy number is also significantly decreased in advanced stage IDC, *p*-value = 0.005. The gene rank for BDP1 is in the top 3% (547) of 18,823 measured genes in IDC, advanced stage. (**C**) BDP1 expression is significantly decreased in the Ginestier breast dataset [52], n = 55 samples and correlates with high grade IDC, *p*-value = 6.81 × 10^−4^. The gene rank for BDP1 under-expression is in the top 1% (193) of 19,574 measured genes in IDC, high grade. (**D**) BDP1 expression significantly decreases in the TCGA breast dataset [45,46,47], *n* = 593 samples and *p*-value = 4.66 × 10^−4^. The gene rank for BDP1 under-expression is in the top 1% (120) of 20,423 measured genes in IDC, advanced stage. Bright blue denotes statistically significant BDP1 changes. The Oncomine™ Platform (Thermo Fisher, Ann Arbor, MI, USA) was used for analysis and visualization.

Copy number alterations of specific genes at specific chromosomes have been demonstrated to correlate with clinical outcomes in breast cancer, including a chromosomal loss in 5q13.2–q15 [49]. BDP1 has been mapped to cytogenetic band 5q13.2 and deletions of chromosome 5q1323 have been identified in poorly differentiated IDC [50] and deletions of chromosome 5q13-14 have been associated with triple-negative breast cancer (TNBC) and poor prognosis [51]. Thus, we sought to determine if the observed BDP1 alterations in copy number and mRNA expression noted in Figure 1 correlate with IDC grade and stage. We queried the Curtis breast dataset (*n =* 1992) [40] to determine if changes in BDP1 copy number correlated with IDC grade. Figure 2A demonstrates that decreased BDP1 copy number negatively correlates with IDC grade, *p =* 2.62 × 10^−19^. BDP1 copy number is also significantly decreased in advanced stage IDC, *p =* 0.005, Figure 2B. Next, we examined BDP1 mRNA expression in grade and stage of IDC patients. In Figure 2C, BDP1 mRNA expression is significantly decreased in the Ginestier breast dataset (*n =* 55) [52], and correlates with high grade IDC, *p =* 6.81 × 10^−4^. BDP1 mRNA expression is significantly decreased in the TCGA breast dataset (*n =* 593) [45,46,47], *p*-value = 4.66 × 10^−4^. We further analyzed BDP1 expression and breast cancer substage using the GEIPA platform [53], Supplementary Figure S1. We examined TP53, BRCA1, and BRCA2 in the breast cancer substage analysis because these genes are frequently altered in sporadic and hereditary breast cancer. BDP1, BRCA1, and BRCA2 expression were significantly altered, whereas TP53, frequently mutated in breast cancer, was not significantly altered, as shown in Figure 2D. These data suggest BDP1 alterations negatively correlate with grade and stage in IDC patients.

### 3.3. BDP1 Alterations Negatively Correlate with Clinical Outcomes in IDC

**Figure 3:**
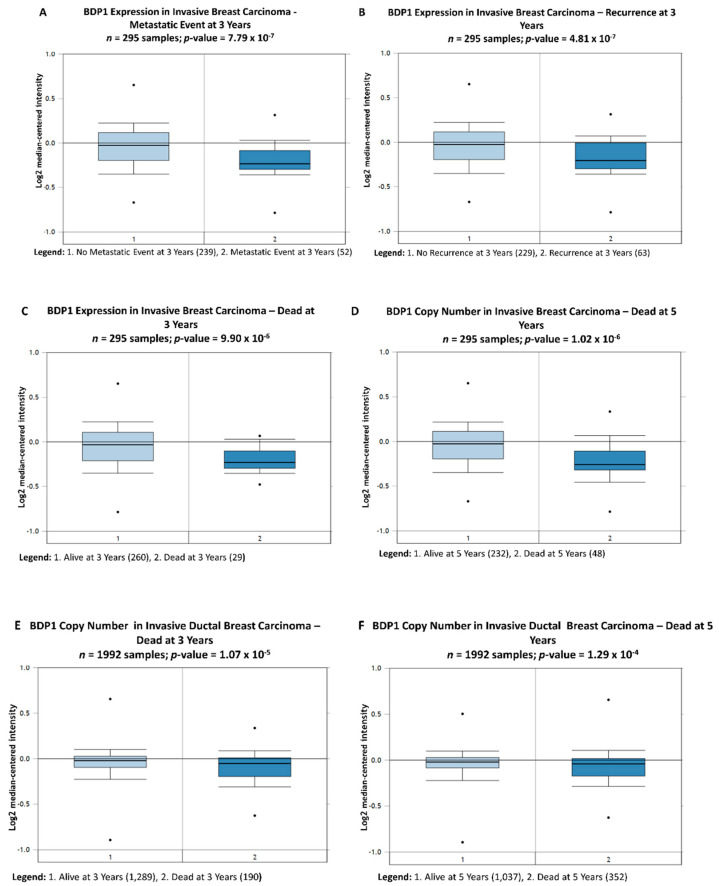
BDP1 alterations negatively correlate with clinical outcomes. Queries of the vandeVijver Breast dataset [48], *n* = 295 samples, identified significant decreases in BDP1 mRNA expression correlates with clinical outcomes in invasive breast cancer (IBC). (**A**) Comparison of nonmetastatic and metastatic events, at three years; BDP1 mRNA expression is significantly decreased (−1.115-fold change), *p*-value = 7.79 × 10^−7^; the gene rank is in the top 1% (47) of 14,719 measured genes in IBC. (**B**) BDP1 mRNA expression is significantly decreased (−1.110-fold change), *p*-value = 4.81 × 10^−7^, in patients with recurring IBC at three years; the gene rank is in the top 1% (82) of 14,719 measured genes. (**C**) BDP1 mRNA expression is significantly decreased (−1.106-fold change), *p*-value = 9.90 × 10^−6^ in patients who died at three years; the gene rank is in the top 1% (110) of 14,719 measured genes in IBC. (**D**) BDP1 mRNA expression is significantly decreased (−1.125-fold change), *p*-value = 1.02 × 10^−6^, in patients with IBC and died at five years; the gene rank is in the top 1% (93) of 14,719 measured genes. (**E**) BDP1 copy number is decreased (−1.038-fold change), *p*-value = 1.07 × 10^−5^, in patients with recurring IDC at three years; the gene rank is in the top 4% (611) of 18,823 measured genes in the Curtis breast dataset [40]. (**F**) BDP1 copy number is decreased (−1.025-fold change), *p*-value = 1.29 × 10^−4^, in patients with recurring IDC at five years; the gene rank is in the top 5% (921) of 18,823 measured genes in the Curtis breast dataset [40]. Bright blue denotes statistically significant BDP1 changes. The Oncomine™ Platform (Thermo Fisher, Ann Arbor, MI, USA) was used for analysis and visualization.

Figure 2 demonstrates that BDP1 copy number and mRNA expression negatively correlate with high grade and advanced stage IDC. Thus, we sought to determine if BDP1 copy number and mRNA expression correlate with clinical outcomes. A query of the vandeVijver breast dataset (*n =* 295) [48] identified decreases in BDP1 mRNA expression that correlate with clinical outcomes in IBC, Figure 3.

Figure 3A compares nonmetastatic and metastatic events at three years. BDP1 mRNA expression is significantly decreased (−1.115-fold change, *p =* 7.79 × 10^−7^) in patients having a metastatic event at three years. BDP1 mRNA expression is significantly decreased (−1.110-fold change, *p =* 4.81 × 10^−7^) in patients with recurring IBC at three years, as shown in Figure 3B. Next, we looked at BDP1 alterations and survival at three and five years. In Figure 3C, BDP1 mRNA expression is significantly decreased (−1.106-fold change, *p*-value = 9.90 × 10^−6^) in patients who died at three years. BDP1 mRNA expression is significantly decreased (−1.125-fold change, *p =* 1.02 × 10^−6^) in patients with IBC who died at five years, as shown in Figure 3D. We next sought to determine if BDP1 copy number correlates with clinical outcomes. Figure 3E provides evidence that BDP1 copy number is decreased (−1.038-fold change, *p =* 1.07 × 10^−5^) in patients with recurring IDC at three years. Additionally, BDP1 copy number is decreased (−1.025-fold change, *p =* 1.29 × 10^−4^) in patients with recurring IDC at five years, as shown in Figure 3F. Taken together, Figure 3 demonstrates that BDP1 alterations negatively correlate with clinical outcomes. The negative correlation between BDP1 copy number and mRNA expression with clinical outcomes in Figure 3 led us to question whether BDP1 copy number and expression levels correlated with known breast cancer biomarkers and clinical outcomes.

### 3.4. BDP1 and Steroid Hormone Expression Correlate with Clinical Outcomes

**Figure 4:**
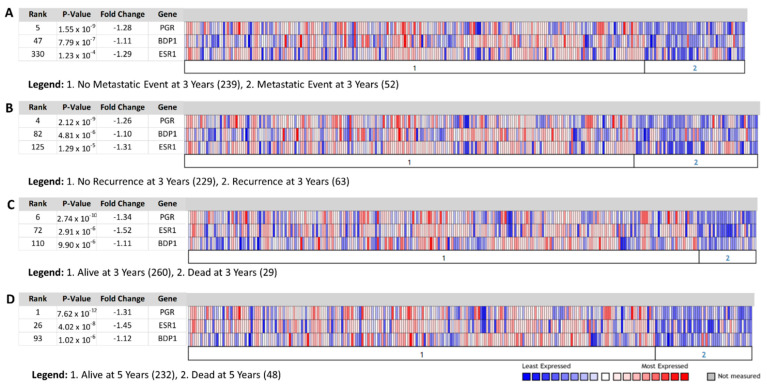
BDP1, progesterone receptor (PR), and estrogen receptor (ESR1) expression correlated with clinical outcomes. We queried the vandeVijver breast dataset [48], *n =* 295 samples, for changes in BDP1 and key breast cancer biomarkers (PR and ESR1) expression and clinical outcomes: (**A**) metastatic event at three years, (**B**) recurrence at three years, death at three (**C**) and five (**D**) years. [40]. Fold-change, *p*-value, and gene rank of 14,719 measured genes in IBC are denoted. The Oncomine™ Platform (Thermo Fisher, Ann Arbor, MI, USA) was used for analysis and visualization.

The estrogen receptor (ER) and progesterone receptor (PR) steroid receptors were the first biomarkers classified in breast cancer [54]. ER-negative breast cancers are more likely to be of higher grade; patients tend to have a decreased overall survival depending on menopausal and lymph node status [55]. In postmenopausal women, a negative ER status is associated with recurrence [56]. PR negative breast cancer patients tend to show cancer recurrence or die within the first five years [57]. Thus, we evaluated if BDP1 mRNA expression correlates with steroid receptor expression and clinical outcomes (Figure 4). Fold-change, *p*-value, and gene rank are noted in Figure 4 legend. In Figure 4A, we compared BDP1, ER (gene symbol ESR1), and PR (gene symbol PGR) mRNA expression in metastatic breast cancer occurring three-year post-diagnosis. Figure 4A suggests BDP1 expression correlates with steroid receptors’ expression in patients with metastatic IBC. Next, we looked at breast cancer recurrence three years post initial diagnosis, as shown in Figure 4B. BDP1, ER, and PR expression was significantly decreased in patients whose breast cancer reoccurred three years after the initial diagnosis. Further, BDP1, ER, and PGR expression correlated with death after three years (Figure 4C) and five years (Figure 4D). Together, the data in Figure 4 suggest BDP1 expression correlates with expression changes to the PR and ER breast cancer biomarkers, and clinical outcomes.

### 3.5. BDP1 Is Altered in Triple-Negative Breast Cancer (TNBC)

**Figure 5:**
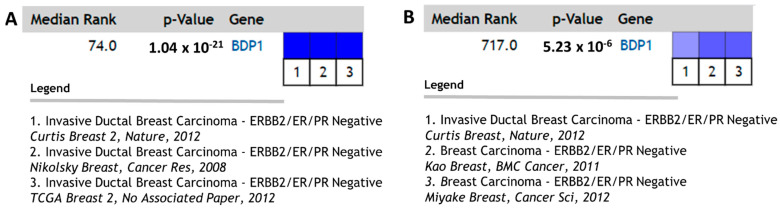
BDP1 is altered in triple-negative breast cancer. (**A**) BDP1 copy number decreased in triple-negative breast cancer, *p =* 1.04 × 10^−21^ (gene rank 74), *n =* 3785 samples [40,44,47]. (**B**) BDP1 expression is decreased in triple-negative breast cancer, *p =* 5.23 × 10^−6^ (gene rank 717), *n =* 2434 samples [40,42,43]. The Oncomine™ Platform (Thermo Fisher, Ann Arbor, MI, USA) was used for analysis and visualization.

BDP1 mRNA expression correlates with ER and PR mRNA expression in the context of clinical outcomes measured in Figure 4. Approximately 15% of IBC is characterized by low expression of ER, PR, and the human epidermal growth factor receptor 2 (HER2) and is classified as TNBC [58]. TNBC is molecularly diverse and disproportionately affects younger women with poor clinical outcomes [59]. BDP1, PR, and ER expression are significantly decreased in the breast cancer datasets queried and negatively correlate with clinical outcomes, as shown in Figure 4. As a result, we speculated if BDP1 is altered in TNBC. We queried the TNBC breast cancer datasets available in the Oncomine™ Platform to identify BDP1 copy number and mRNA expression alterations in TNBC, as shown in Figure 5. BDP1 copy number is significantly decreased in triple-negative breast cancer, *p =* 1.04 × 10^−21^, *n =* 3785 samples across three datasets [40,44,47], as shown in Figure 5A. Approximately 70% of TNBCs has been demonstrated to undergo deletions spanning the long arm of chromosome, including 5q13-14 [51], and BDP1 is located at 5q13.2. BDP1 mRNA expression is decreased in TNBC, *p =* 5.23 × 10^−6^, *n =* 2434 samples across three datasets [40,42,43], as shown in Figure 5B. We cannot rule out the possibility that the decreased BDP1 mRNA expression may be due, in part, to the decrease in ER expression. We queried the Eukaryotic Promoter Database (https://epd.epfl.ch, accessed January–February 2022) [60] and identified four putative ER binding sites at −976, −785, −711, and −535, *p =* 0.001, in the BDP1 promoter that may regulate BDP1 expression.

### 3.6. BDP1 mRNA Expression Is Increased by Breast Cancer Chemotherapeutics

In this study, we demonstrate BDP1 copy number and mRNA expression decrease in IDC (Figure 1). BDP1 alterations negatively correlate high grade and advanced stage (Figure 2). Additionally, BDP1 alterations negatively correlate with clinical outcomes (Figure 3), correlate with ER and PR expression in IBC (Figure 4), and significantly decrease in TNBC (Figure 5). Together, these data prompted us to determine if available public datasets provide insight into whether BDP1 is a potential therapeutic target in breast cancer. Using Oncomine, we performed a perturbation analysis of breast cancer datasets available to potentially identify chemotherapeutic agents regulating BDP1 mRNA expression. Based on the statistically significant decrease in BDP1 mRNA expression (*p* = 1.04× 10^−21^) and significant gene rank (74) in TNBC, across three studies and *n =* 3785 samples [40,44,47] (Figure 5), we queried for chemotherapeutics known to be effective in treating TNBC [61,62,63]. TNBC can be further classified into subtypes, including basal-like TNBC.

**Figure 6:**
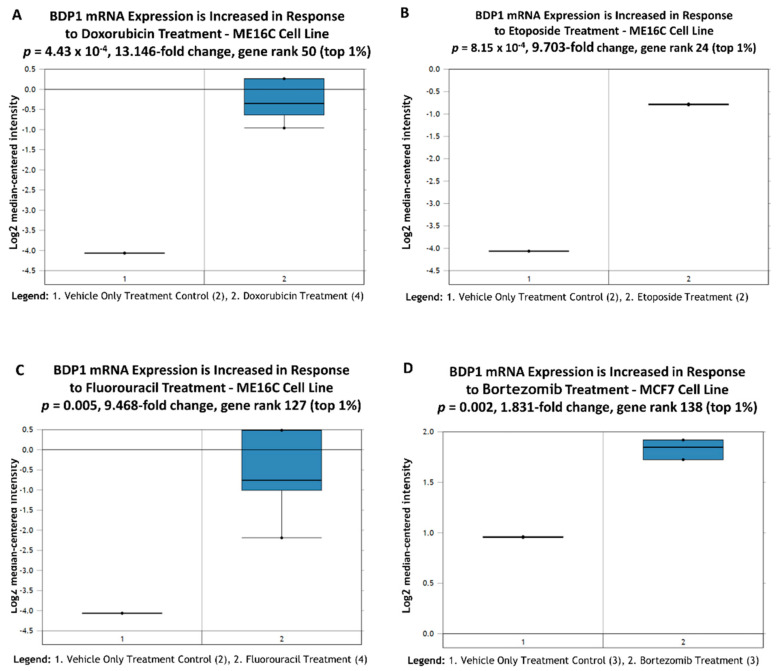
Breast cancer chemotherapeutics increase BDP1 mRNA expression. A query of the Troester [35] and Nickeleit [36] cell line datasets for BDP1 mRNA expression in ME16C and MCF7 cells in response to (**A**) doxorubicin, (**B**) etoposide, (**C**) fluorouracil, or (**D**) bortezomib treatment. *p*-values, fold-change, and gene rank are indicated in the figure. Detailed methods are provided in the published papers by Troester et al. [35] and Nickeleit et al. [36]. Bright blue denotes statistically significant BDP1 changes. The Oncomine™ Platform (Thermo Fisher, Ann Arbor, MI, USA) was used for analysis and visualization.

We first queried the Troester [35] cell line dataset. We noted that BDP1 mRNA expression is significantly increased (*p =* 4.43 × 10^−4^) in the basal-like hTERT-immortalized human mammary epithelial (HME) cell line ME16C cells treated with doxorubicin (DOX), as shown in Figure 6A. DOX intercalates into DNA strands, inhibiting DNA topoisomerase II, ultimately inducing apoptosis [64]. Dox-based chemotherapy is one of the common treatments for TNBC but identifying biomarkers capable of predicting whether TNBC patients will respond to DOX remains challenging and ongoing. Recently, it has been demonstrated that DOX inhibits cell proliferation by activating cAMP response element binding protein 3-like 1 (CREB3L1) and that CREB3L1 expression determines DOX sensitivity [65]. A query of the Eukaryotic Promoter Database (https://epd.epfl.ch, accessed January–February 2022) [60] identified three putative CREB3L1 binding sites at −589, −394, and −392, *p =* 0.001, in the BDP1 promoter, suggesting a potential mechanism for regulation of BDP1 expression in DOX treated ME16C cells. Figure 6A prompted us to query additional TNBC chemotherapeutics potentially regulating BDP1 expression. BDP1 mRNA expression is significantly increased 9.703-fold (*p =* 8.15 × 10^−4^) in etoposide (ET) treated ME16C cells, as shown in Figure 6B. ET is a chemotherapeutic used to treat TNBC and induces double-stranded DNA breaks [61]. In Figure 6C, we queried for changes to BDP1 mRNA expression in response to fluorouracil treatment (5-FU), one of the oldest chemotherapeutics used to treat breast cancer using the Troester [35] cell line dataset. ME16C cells treated with 5-FU increased BDP1 mRNA expression 9.468-fold, gene rank 127 (top 1%), *p =* 0.005. In Figure 6D, we queried for changes to BDP1 mRNA expression in response to bortezomib treatment of MCF-7 cells in the Nickeleit [36] cell line dataset. Bortezomib is a proteasome inhibitor inducing apoptosis in TNBC cell lines by downregulating CIP2A-dependent p-Akt, demonstrated to be associated with more aggressive breast cancers [63]. BDP1 mRNA expression 1.831-fold, gene rank 138 (top 1%), *p =* 0.002, as shown in Figure 6D. It is well established that the PI3K/Akt/mTOR/S6K pathway regulates RNA polymerase III transcription through TFIIIB [13]. However, it has not been determined if the TFIIIB subunit BDP1 is directly regulated by the PI3K/Akt/mTOR/S6K pathway. Together, Figure 5 and Figure 6 suggests a larger study of potential alterations in BDP1 expression in TNBC patients is warranted.

## 4. Discussion

To the best of our knowledge, this is the first study to correlate BDP1 alterations with clinical outcomes in breast cancer. The functional genomics approach to investigating BDP1 alterations in breast cancer is significant. The BDP1 gene encodes a 2250 amino acid protein with a predicted molecular weight of 250 kilodaltons and characterized in vitro characterization [8,9]. Early in vitro characterization of BDP1 has achieved using truncated forms of BDP1 to elucidate structural and RNA pol III promoter binding information [66]. The size of BDP1 has hindered researchers from working on full-length BDP1 in vitro to the extent in vitro characterization has progressed on other TFIIIB subunits. Advances in microarray and RNA-sequencing have provided a more focused approach to studying TFIIIB activity in cancer, specifically with these analyses performed using clinical samples [6,25,34,67].

Herein, we report that BDP1 copy number (*n =* 1602; *p =* 8.03 × 10^−9^) and mRNA expression (*n =* 130; *p =* 0.002) are specifically decreased in patients with invasive ductal carcinoma (IDC), as shown in Figure 1. BDP has been cytogenetically mapped to 5q13.2., and deletions of chromosome 5q13-23 have been identified in poorly differentiated IDC [50]. Chromosome 5q13-14 have been associated with triple-negative breast cancer (TNBC) and poor prognosis [51]. Hence, we examined if BDP1 alterations correlated with clinical outcomes in breast cancer. BDP1 copy number negatively correlated with high grade (*n =* 1992; *p =* 2.62 × 10^−19^) and advanced stage (*n =* 1992; *p =* 0.005), as shown in Figure 2. In Figure 2, we also demonstrate that BDP1 mRNA expression negatively correlated with high grade (*n =* 55; *p =* 6.81 × 10^−4^) and advanced stage (*n =* 593; *p =* 4.66 × 10^−4^) IDC. Decreased BDP1 expression correlated with clinical outcomes, as shown in Figure 3 (*n =* 295 samples): a metastatic event at three years (*p =* 7.79 × 10^−7^) and cancer reoccurrence at three years (*p =* 4.81 × 10^−7^) in patients with invasive breast cancer. Decreased BDP1 mRNA correlates with patient death at three (*p =* 9.90 × 10^−6^) and five (*p =* 1.02 × 10^−6^) years.

BDP1 mRNA expression correlates with ER and PR receptor expression in patients with metastatic breast cancer occurring three-year post-diagnosis (Figure 4A), patients with breast cancer recurring three years post initial diagnosis (Figure 4B) and correlated with death after three years (Figure 4C) and five years (Figure 4D). Together, the data in Figure 4 suggest BDP1 expression correlates with expression changes to the PR and ER breast cancer biomarkers and clinical outcomes.

Roughly 15% of IBC is characterized by low ER, PR, and HER2 expression and is classified as TNBC [58]. BDP1, PR, and ER expression are significantly decreased in the breast cancer datasets queried and negatively correlate with clinical outcomes, as shown in Figure 4. As a result, we speculated if BDP1 is altered in TNBC. BDP1 copy number is significantly decreased in triple-negative breast cancer, *p =* 1.04 × 10^−21^, *n =* 3785 samples across three datasets [40,44,47], as shown in Figure 5A. Approximately 70% of TNBCs have been demonstrated to undergo deletions, including 5q13-14 [51], and BDP1 is located at 5q13.2. BDP1 mRNA expression is decreased in TNBC, *p =* 5.23 × 10^−6^, *n =* 2434 samples across three datasets [40,42,43], as shown in Figure 5B. We cannot rule out the possibility that the decreased BDP1 mRNA expression may be due, in part, to the decrease in ER expression. We identified four putative ER binding sites at −76, −785, −711, and −535, *p =* 0.001 [60], in the BDP1 promoter that may regulate BDP1 expression. We did not identify any putative PR binding sites in the BDP1 promoter.

BDP1 mRNA expression is increased by the breast cancer chemotherapeutics doxorubicin (13.146-fold increase; *p =* 4.43 × 10^−4^), etoposide (9.703-fold increase; *p =* 8.15 × 10^−4^), fluorouracil (9.468-fold increase; *p =* 0.005), and bortezomib (1.831-fold increase; *p =* 0.002) in well-studied breast cancer cell lines [35,36], as shown in Figure 6. Together, the data presented in this study suggest that BDP1 may be a novel target for therapeutic intervention for patients with breast cancer, and larger studies are warranted.

## 5. Conclusions

Breast cancer accounts for 30% of all new cancer diagnoses in the United States. The most common type of breast cancer is invasive breast cancer. A hallmark trait of breast cancer is uncontrolled cell growth due to genetic alterations. TFIIIB-mediated RNA polymerase III transcription is specifically deregulated in human cancers. The TFIIIB BDP1 subunit is not well characterized in human cancer. The objective of this study was to analyze publicly available clinical cancer datasets to determine if BDP1 alterations correlate with clinical outcomes in available breast cancer datasets. BDP1 copy number and expression negatively correlate with breast cancer outcomes, including stage, grade, and mortality. Specifically, we report that BDP1 copy number (*n =* 1602; *p =* 8.03× 10^−9^) and mRNA expression (*n =* 130; *p =* 0.002) are decreased in patients with invasive ductal carcinoma (IDC). In IDC, BDP1 copy number negatively correlated with high grade (*n =* 1992; *p =* 2.62 × 10^−19^) and advanced stage (*n =* 1992; *p =* 0.005). BDP1 mRNA expression negatively correlated in high grade (*n =* 55; *p =* 6.81 × 10^−4^) and advanced stage (*n =* 593; *p =* 4.66 × 10^−4^) IDC. BDP1 mRNA correlates with patient death at three (*p =* 9.90 × 10^−6^) and five (*p =* 1.02 × 10^−6^) years. Also, BDP1 copy number decreased in TNBC (*n =* 3786; *p =* 1.04 × 10^−21^). Both BDP1 copy number (*n =* 3785; *p =* 1.0 × 10^−14^) and mRNA expression (*n =* 2434; *p =* 5.23 × 10^−6^) are altered in TNBC. Finally, BDP1 mRNA expression is increased by the breast cancer chemotherapeutics doxorubicin (13.146-fold increase; *p =* 4.43 × 10^−4^), etoposide (9.703-fold increase; *p =* 8.15 × 10^−4^), fluorouracil (9.468-fold increase; *p =* 0.005), and bortezomib (1.831-fold increase; *p =* 0.002) in well-studied breast cancer cell lines. Together, the data presented suggest a role for BDP1 alterations in invasive breast cancer. 

## Figures and Tables

**Table 1:** Datasets used in study. Hyperlinks to datasets are provided.

Dataset	Study Description and Link to Dataset	Reference
Barretina	The Cancer Cell Line Encyclopedia (CCLE), consisting of 947 human cancer cell lines encompassing 36 tumor types, used genomic technology platforms for characterization. Of the 947 human cancer cell lines, 56 cell lines represented breast cancer types. Link to dataset: http://www.ncbi.nlm.nih.gov/geo/query/acc.cgi?acc=GSE36138, accessed January 2020–January 2022.	[39]
Curtis 2	A total of 1992 primary breast tumors were studied using an integrated genomic and transcriptomic analysis. Overall, 997 of the 1992 represent a discovery set of primary tumors and 995 of the 1992 tumors were a validation set from the Molecular Taxonomy of Breast Cancer International Consortium (METABRIC). Link to dataset: http://www.ebi.ac.uk/ega/studies/EGAS00000000083, accessed January 2020–January 2022.	[40]
Esserman	The I-SPY 1 trial (investigation of serial studies to predict your therapeutic response with imaging and molecular analysis) collected invasive breast cancers from patients with tumor size >3 cm and patients with T4 or inflammatory disease. Patients were evaluated for specific biomarker profiles to understand chemotherapy response and recurrence-free survival. Then, 130 out of the total 221 patients eligible for analysis were used in this report. Link to dataset: https://www.ncbi.nlm.nih.gov/geo/query/acc.cgi?acc=GSE22226, accessed January 2020–January 2022.	[41]
Kao	This study analyzed 327 breast cancer samples for gene expression profiles resulting in identification of molecular subtypes with distinct molecular and clinical characteristics. Link to dataset: http://www.ncbi.nlm.nih.gov/geo/query/acc.cgi?acc=GSE20685, accessed January 2020–January 2022.	[42]
Miyake	This study, primary breast cancer patients, stage II-III, treated with neoadjuvant 5-fluoruracil/epirubicin/cyclophosphamide (P-FEC) were analyzed (*n* = 123). Link to dataset: http://www.ncbi.nlm.nih.gov/geo/query/acc.cgi?acc=GSE32646, accessed January 2020–January 2022.	[43]
Nickeleit	MCF-7 breast cancer cells were treated with bortezomib, a proteasome inhibitor. Link to dataset: http://www.ncbi.nlm.nih.gov/geo/query/acc.cgi?acc=GSE8565, accessed January 2020–January 2022.	[36]
Nikolsky	A total of 191 breast tumors (154 primary tumors and 37 breast cancer cell lines) were characterized for copy number alterations. Link to dataset: https://portals.broadinstitute.org/tcga/home, accessed January 2020–January 2022.	[44]
TCGA	The Cancer Genome Atlas Program (TCGA) characterized over 20,000 primary cancer and normal samples for 33 cancer types. For this study, we utilized the invasive ductal carcinoma datasets (*n* = 1602). Link to dataset: http://tcga-data.nci.nih.gov/tcga/, accessed January 2020–January 2022.	[45,46,47]
Troester	This study utilized ME16C and HME-CC cells (basal-like hTERT-immortalized HME cell lines), MCF-7 and ZR-75-1 cells to identify genes that showed differential expression between DOX- and 5FU-treatment to determine if there were toxicant-specific gene expression patterns. Link to dataset: http://www.ncbi.nlm.nih.gov/geo/query/acc.cgi?acc=GSE1647, accessed January 2020–January 2022.	[35]
vandeVijver	A total of 295 primary invasive breast carcinomas were analyzed for gene-expression signatures associated with poor prognosis or good prognosis. Breast dataset invasive breast carcinoma. Link to dataset: https://www.ncbi.nlm.nih.gov/geo/query/acc.cgi?acc=GSE2845, accessed January 2020–January 2022.	[48]

## Data Availability

The present study used publicly available datasets. Hyperlinks to datasets utilized are provided in Table 1. Data analysis was performed using Oncomine Research Edition, retired on 17 January 2022.

## Supplementary Materials

The following supporting information can be downloaded at: https://www.mdpi.com/article/10.3390/cancers14071658/s1, Figure S1: BDP1 mRNA expression correlates with substages in breast cancer.
