# Supplementary material for: BDP1 Alterations Correlate with Clinical Outcomes in Breast Cancer
Source: Cancers (Basel). 2022 Mar 25;14(7):1658. doi: 10.3390/cancers14071658 (PMC8996959; doi:10.3390/cancers14071658)
Supplement: Supplementary file 1 [file cancers-14-01658-s001.zip › cancers-1618265-supplementary.pdf]

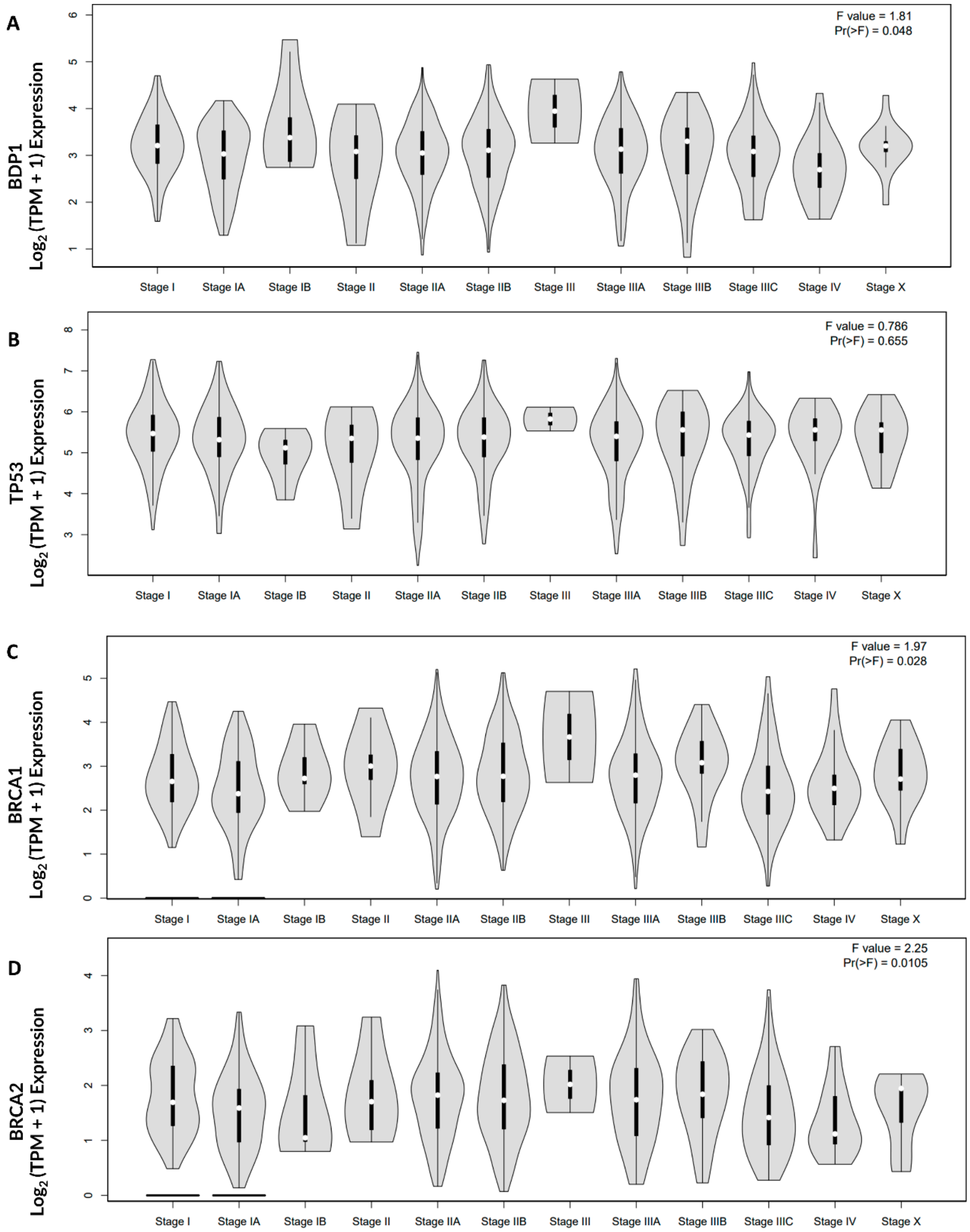

Supplemental Figure S1. BDP1 mRNA expression correlates with substages in breast cancer. We queried the Gene Expression Profiling Interactive Analysis (GEPIA) (1) to evaluate if BDP1 mRNA expression correlates with sub-stages stage in breast cancer samples, compared to control, from the Cancer Genome Atlas (TCGA) and Genotype-Tissue Expression (GTEx) project (A) BDP1, (B) TP53, (C) BRCA1, and (D) BRCA2. The matched normal analysis was performed using TCGA tumors vs TCGA normal

+ GTEx normal. Stage X are samples from breast cancer patients whose cancer substage was unclassified. $Log_2(TPM + 1)$ transformed expression data was used for plotting. One-way ANOVA analysis was performed. F and Pr(>F) values are denoted for each gene.

Reference:

1. Tang, Z. et al. (2017) GEPIA: a web server for cancer and normal gene expression profiling and interactive analyses. Nucleic Acids Res, 10.1093/nar/gkx247.
